# Chipless RFID Label with Identification and Touch-Sensing Capabilities

**DOI:** 10.3390/s21144862

**Published:** 2021-07-16

**Authors:** Rahul Unnikrishnan, Olivier Rance, Nicolas Barbot, Etienne Perret

**Affiliations:** 1 LCIS Laboratory, Grenoble INP, Université Grenoble Alpes, Grenoble INP, LCIS, F-26000 Valence, France; Rahul.Unnikrishnan@lcis.grenoble-inp.fr (R.U.); Olivier.Rance@lcis.grenoble-inp.fr (O.R.); nicolas.barbot@lcis.grenoble-inp.fr (N.B.); 2 Institut Universitaire de France, 75005 Paris, France

**Keywords:** chipless RFID, back scattering, gesture recognition

## Abstract

This article presents a 14-bit chipless RFID label which, in addition to classical identification feature, can be used as decimal numeric keypad, allowing the deployment of secure access control applications. A low-cost single layer label comprising 10 RF loop scatterers is used to code information in the frequency domain. In addition, each resonator is associated to a digit in the decimal number system, and the difference in the spectrum caused by the touch event is exploited for the detection of each key pressing. The shape of the resonators has been carefully selected to be both highly resonant and to show high sensitivity to the presence or absence of the human finger. The concept is validated by measurements in an office environment using an FCC compliant low-cost chipless reader and microstrip vivaldi antennas. Simple detection algorithms are proposed for both identification and touch sensing in real environment.

## 1. Introduction

Radio frequency identification (RFID) is a wireless counterpart of barcode identification techniques. RFID has come into picture as a serious contender to the conventional optical identification techniques because of the advantages, which is unique to the system by the usage of radio waves [1]. In the last few decades, serious research has been carried out around the globe to increase the performance of the system, including the reading distance or the cost of the tag, which is one of the primary parameters when it comes to the mass production of the system. The main hindrance in reducing the manufacturing cost is due to the integrated circuit associated with the system.

Chipless RFID technology has come to life as a solution to overcome the hurdle of fabrication cost faced by conventional chipped RFID tags. This non-chip identification solution with its advantages, in terms of its operating principles, has attracted a lot of researchers around the globe. Though, the chipless RFID technology has begun as an identification technology, researchers are now moving towards giving it additional functionalities and to use it as a sensor device. Thus, the new paradigm of “smart chipless electronic label” is born [2], with the objective to replace some current applications based on classical chipped devices. This concept opens up a new horizon in wireless communication by providing simple tools to remotely interact with objects.

In today’s era of IoT devices, humans have been continuously interacting with the devices like no other time before. The IoT trend also fueled the researchers to find a way to interact the devices wirelessly in a cost-effective manner. Since the past decade, the RFID technology has started to put its footprint in the form of human gesture recognition sensor to interact with various devices. Thorough research has been conducted to find the effects of human body interaction on RFID devices [3] and the impact in the real-life scenario is also studied [4,5,6,7]. In this context, it becomes of great interest to show that a chipless tag, in addition to being an identification device, can be used as a touch sensor to convey useful information to the reader. In this scenario, resonators initially used for coding information also become the base element for a low-cost user interface directly integrated within the label.

The studies are already in thrust for creating human gesture recognizer using radar techniques [8,9,10,11,12,13] and RF touchpads which can be printed on a flexible substrate, like in [14,15,16]. Examples of remote-control human computer interaction (HCI) based on chipped RFID technology are also available in literature, either for low-cost control panels [17] or for inferring human activities from interactions with objects [18]. Although very promising, this kind of HCI requires a chip for each key.

The concept of remote-control interface using chipless RFID has been first introduced in [19] and a second implementation was performed in [20]. The authors show that classical chipless tags working on ultra-wide band (UWB) frequency and based on RF encoding particles (REP) can be used as touch sensors. Although promising, these two contributions are limited by the cost of both the tag and the global system which make them irrelevant in comparison with commercially available technologies. Compared to a barcode, in both cases, the tag is realized on expensive grounded RF substrate and comprises only six resonators (i.e., not compatible with a common decimal numeric keypad with digit 0 to 9). The measurements are performed using expensive laboratory material (VNA and antennas). Furthermore, only the capability of the tag to be used as a touch sensor is discussed in [19,20]. The touch-sensing systems mentioned in [19,20] are also limited as the hand comes between the reader and tag when touch event occurs which considerably reduces the reading quality such as the reading distance or the reading rate. This makes the measuring setup with stringent requirements which can be a disadvantage when it comes to the implementation of the system in the real world. Even though the RF approaches, which were mentioned earlier, give promising performances [8,9,10,11,12,13], the question of identification remains un-addressed.

In this article, for the first time, we are trying to exploit the possibility of a chipless label to use as an identifier-touch sensor in a realistic scenario with a low-cost tag and reading setup (using microstrip antennas and a low-cost chipless reader [21]). A single-layer label (no ground plane) was designed and realized on PET substrate. It is composed of ten loop resonators spread over the 3–5.6 GHz band achieving a 14-bit coding capacity. The resonators were designed to possess high Q factor and radar cross section (RCS) levels in order to increase the probability of human touch detection. Both identification and touch-sensing capabilities were evaluated from measurement. A comparative study was also conducted to evaluate the performance of the simulated and measured tag. The practical limitations of the measuring set-up in [19,20] are addressed and a better measuring setup is proposed by using single-layer tag which can be touched on both sides. The touching was performed on the back side of the tag which reduced both the interaction of adjacent resonators by the human hand and the possibility of direct interaction of RF waves. The financial viability parameter was also considered during the system design, both on the tag side as well as the reader side in order to achieve a system form factor comparable to an RFID system available on the market. The proposed system has unique ID and remotely detects the human touch event from the signal backscattered from the tag with a total form factor which is comparable to the classical chipped RFID reader system.

The article first presents the principle of operation of the system and the selected REP in Section 2. The effect of the finger is investigated numerically in Section 3 and experimental validation and results are presented in Section 4.

## 2. Design

### 2.1. Principle of Operation

**Figure 1:**
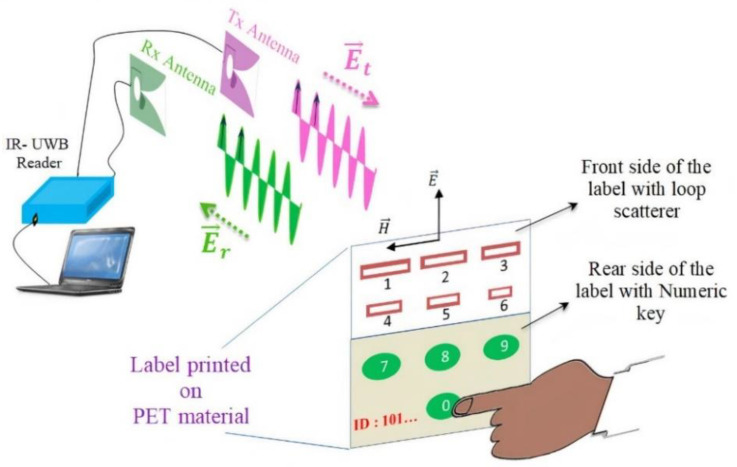
System architecture of wireless numeric keypad based on Chipless RFID.

The chipless RFID system is represented schematically in Figure 1. It can be seen as a RADAR system for which the target has been specifically designed to be easily distinguishable. A dedicated reader sends a UWB pulse to the label which is composed of resonant loop scatterers. The resonant behavior of the scatterers creates a series of peaks in the spectrum of the backscattered signal which are used to encode information.

When it comes to application, the label reading is separated in two successive steps, corresponding to the decoding of the identifier and then to the detection of a key-pressing.

**Figure 2:**
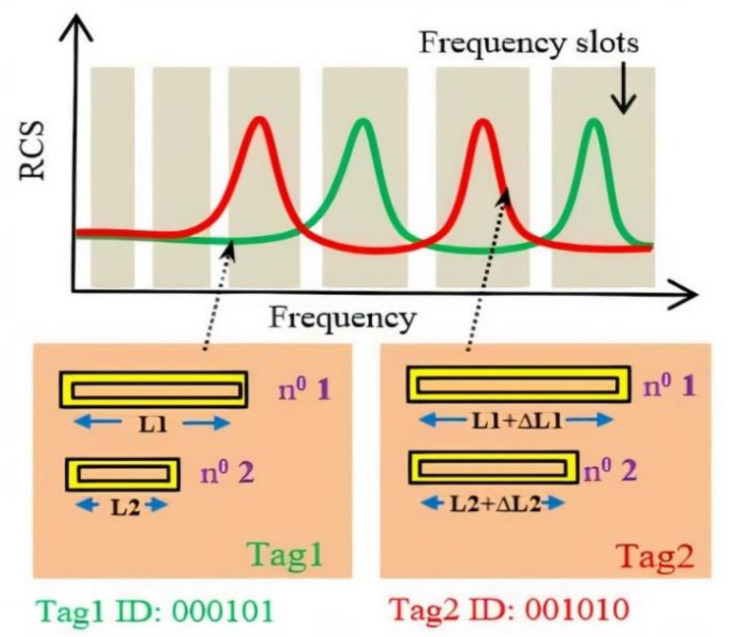
Frequency Position Coding (FPC) technique used in chipless RFID.

In the proposed label, the information is coded using the classic frequency position coding (FPC) technique which presents both high coding capacity and good robustness. The frequency band is divided into N frequency slots with uniform or non-uniform lengths, and the presence or absence of a peak in the slot is used to encode the information (see Figure 2). The resonant frequencies of the resonators can be modified simply by varying the geometrical dimensions of the individual scatterers. The coding capacity is thus limited by the frequency band and the Q factor of the chosen resonators.

**Figure 3:**
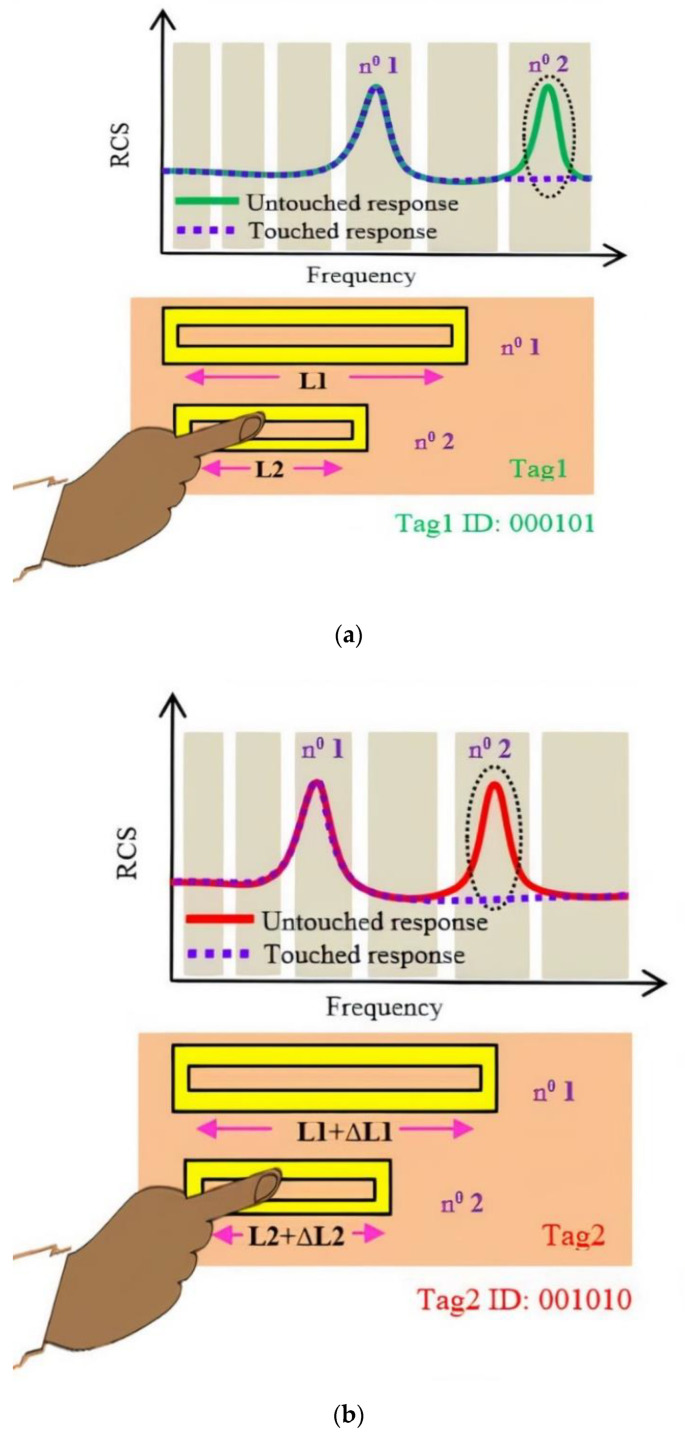
Wireless Numeric keypad technique applied for the tags mentioned in Figure 2: (**a**) Tag1; (**b**) Tag 2.

Once the label identifier is first decoded by the reader, the label can then be used as a wireless numerical keypad (see Figure 3). When the finger is in contact with a resonator, it cancels the corresponding resonance peak which was initially present in the signature of the backscattered signal. The disappearance of the peak at a specific frequency is brought out by a differential measure and is then used as an indicator of which numerical key has been pressed.

### 2.2. Chipless Tag

**Figure 4:**
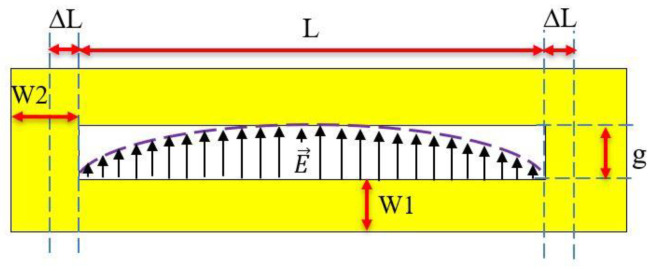
Geometry of the loop scatterer.

Contrary to [19,20], a single-layer label was chosen to be designed (no ground plane) in order to be both compatible with low-cost fabrication process (such as inkjet printing) and to achieve a practical setup where the presence of the hand is not masking the label at the press of a key (see Figure 1). Single-layer labels are much more challenging to design and to measure than the label comprising a ground plane due to lower Q and a higher susceptibility to the environment. The proper selection of the geometry of the tag plays an important role for the design. A series of simulations showed that the loop resonator (Figure 4) presents very good radar cross section (RCS) and Q factor for a single-layer structure while being a good compromise in terms of compactness and easiness of fabrication which is vital for real applications. As it will be shown in the following sections, this REP also has the advantage of having limited couplings and a good sensitivity to the finger presence.

The loop resonators (represented with geometrical parameters in Figure 4) can be modelled as a coplanar stripline of length *L* with short circuits (SC) at both terminations. At radio frequency, the shorted ends depart from ideal SC due to magnetic energy stored behind the termination. This can be modelled by an additional length ∆*L* as in [22], and $L^{\prime }$ is the total effective length (see Equation (1)). The resonant frequency of the loop is then classically obtained from the study of the resonance equation associated to the equivalent circuit [22] which gives

(1) $$L+2\cdot \Delta L=m\cdot \lambda_{g}/2=L^{\prime }$$

where *λg* is the guided wavelength and m is an integer. The resonant frequency of the fundamental mode is given by

(2) $$f_{r}=\frac{c}{2\sqrt{\varepsilon_{ef}}\cdot \left(L+2\Delta L\right)}$$

where *c* is the speed of light in vacuum and *ε_ef_* is the effective permittivity of the coplanar stripline which depends on the substrate characteristics and on the transverse dimensions W1 and g [23]. As represented schematically in Figure 4, at resonance, the electric field stored in vicinity of the tag follows a cosine distribution with zero at the SC boundary conditions and maximum at the center of the loop similar to the TE_10_ mode of a cavity. This ensures an important effect when the finger presses at the middle of the resonator. Although more bulky than other single-layer resonators, such as the C-shape [24], the loop presents the advantage to have a higher RCS level (−14 dBsm at 3.0 GHz). The loop is also less subject to couplings than the C-shape due to the fact that both terminations are SC. The re-radiation pattern of this scatterer follows a sine distribution with respect to elevation angle and presents zero re-radiation in the plane of the tag.

## 3. Full-Wave Simulations

The effect of the finger touching the scatterer were investigated in simulations using time-domain solver of the full-wave commercial software CST Microwave studio. The human finger can be considered as a multilayer lossy medium composed of different tissues (skin, fat, blood, and bone) [25]. Each layer had its own permittivity, conductivity, and density. The hand model used for the simulation was taken from the CST human voxel family. The voxel model was made of 2 to 5 million voxels with a resolution of 2.08 × 2.08 × 8.0 mm^3^ [26]. The permittivity of the tissues was calculated by the Cole-Cole relaxation model. The model used permittivity values of 61.3, 12.4, 5.4, 41.4 and loss tangent values of 0.500, 0.229, 0.186, and 0.418 for blood, bone, fat, and skin, respectively [27,28,29,30]. The resonator used for the simulation had a geometrical length L of 43.8 mm and resonated at 3.05 GHz. The resonator was positioned on a dielectric substrate of a thickness of 0.2 mm and a dielectric constant of 2.6.

**Figure 5:**
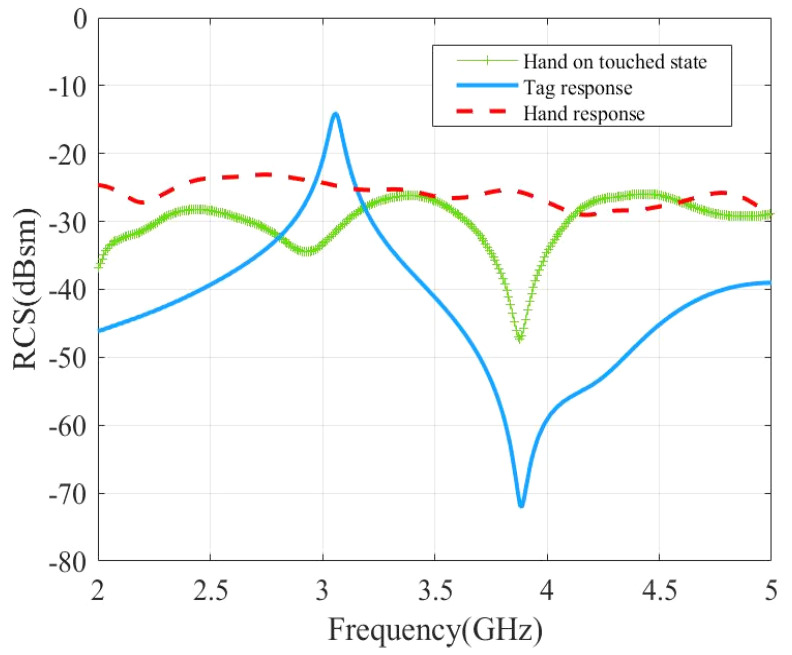
Simulated RCS response of the tag to study the impact of the hand touch event.

A series of three simulations, comprising the loop resonator alone, the hand alone, and the hand touching the loop, were carried out (Figure 5). For the resonator alone, an apex of −14 dBsm was observed at resonance (3.05 GHz), followed by an anti-resonance (pronounced dip of −70 dBsm at 3.9 GHz) which was due to the complex summation between the structural mode and the antenna mode of the tag. The signature associated to the hand alone was non-resonant and showed an RCS level of approximately −25 dBsm on the band 2 GHz to 5 GHz. When the finger was in contact with the loop resonator, simulations showed an RCS reduction of 16 dBsm at the resonance frequency compared to the tag alone. The RCS level was inferior to −25 dB in the whole band and a dip of −45 dB was still observed at 3.9 GHz.

**Figure 6:**
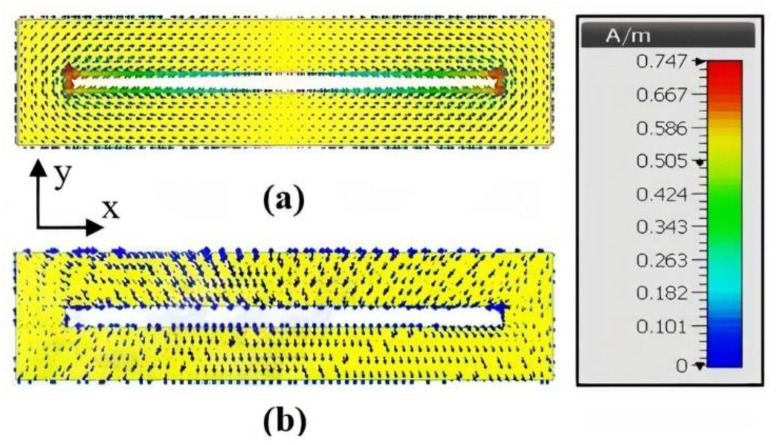
Surface current distribution of the loop scatterer at resonant frequency 3.05 GHz: (**a**) Scatterer is in untouched state; (**b**) finger is in contact with the scatterer.

Surface current distributions of the loop scatterer with and without hand touch are compared in Figure 6. In absence of the hand, the intensity of the current exhibited a sine distribution along the long strips of the loop (x direction) with zero at the center of the loop and maximum at the edges. The current was concentrated on the small arms (y direction) of the loops, for which the distribution was almost constant (infinitesimal dipole), and the current reached a maximum intensity of 0.747 A/m. When the finger was in contact with the loop, the current inside the loop vanishes (0.0163 A/m) and the loop was not resonant anymore which confirms the disappearance of the peak, as observed in Figure 5.

## 4. Analytical Model

**Figure 7:**
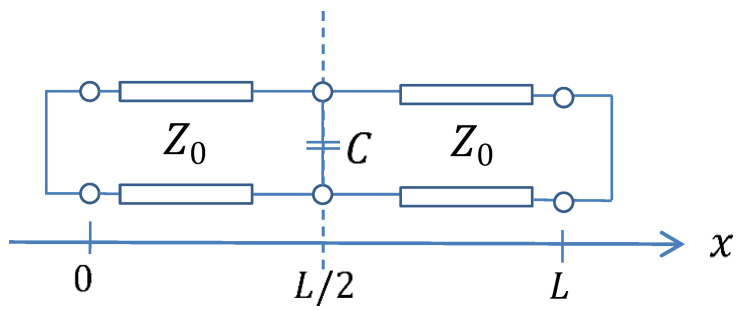
Analytical model for the effect of the human finger (parallel capacitor *C*) when touching the resonator. The loop was modeled by a SC transmission line (TL) of characteristic impedance $Z_{0}$ where a capacitor *C* was considered at the center of the TL.

A simple analytical model was developed to study the mechanism of the impact of the hand as shown in Figure 7. The parameter *L* shows the geometrical length of the resonator and that the effect of the hand is modelled as the parallel capacitor *C* in Figure 7. From this model, the resonance equation of the modified resonator leads to Equation (3).

(3) $$\sin\left(2\pi f_{r}\sqrt{\varepsilon_{ef}}\cdot L^{\prime }/c\right)+2\pi f_{r}\frac{Z_{0}C}{2}\cdot \cos\left(2\pi f_{r}\sqrt{\varepsilon_{ef}}\cdot \frac{L^{\prime }}{c}\right)-2\pi f_{r}\frac{Z_{0}C}{2}=0\;\;\;$$

The $Z_{0}$ in the equation corresponds to the characteristic impedance of the TL, which depends on the value of the transverse geometrical dimensions. For a given loop geometry, it is possible to use Equation (3) to derive the resonant frequency f of the loop with the finger effect. The value of the equivalent capacitance *C* can be calculated using Equation (4) where $f_{r}$ is evaluated from a full wave simulation comprising the tag and the finger.

(4) $$C=\frac{2}{Z_{0}\cdot 2\pi f_{r}}\left[\frac{\sin\left(2\pi f_{r}\sqrt{\varepsilon_{ef}}\cdot \frac{L^{\prime }}{c}\right)}{1-\cos\left(2\pi f_{r}\sqrt{\varepsilon_{ef}}\cdot \frac{L^{\prime }}{c}\right)}\right]$$

**Figure 8:**
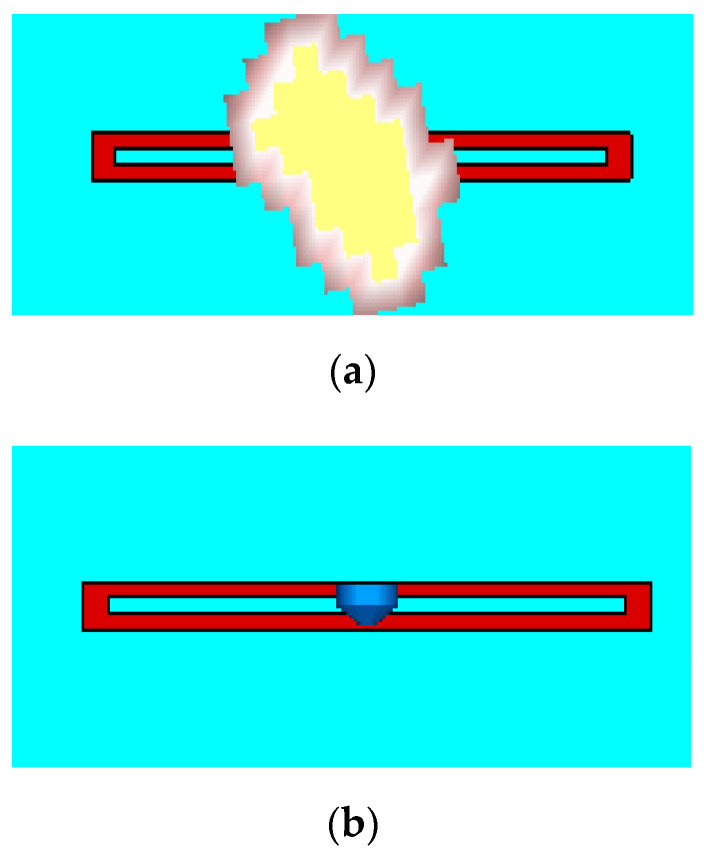
Simulation Structure corresponding to: (**a**) finger touch (only the fingertip was considered in the simulation); (**b**) lumped capacitor in loop structure.

**Figure 9:**
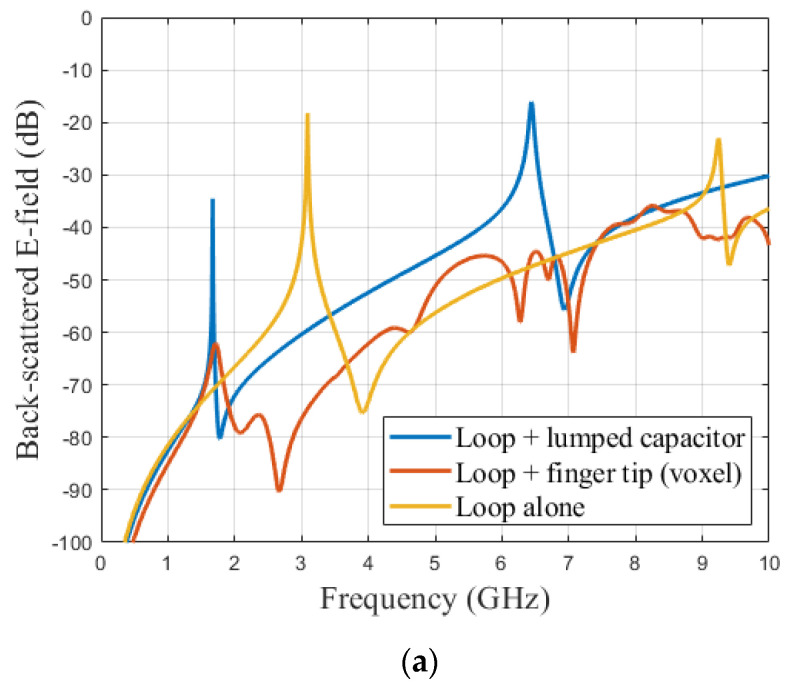

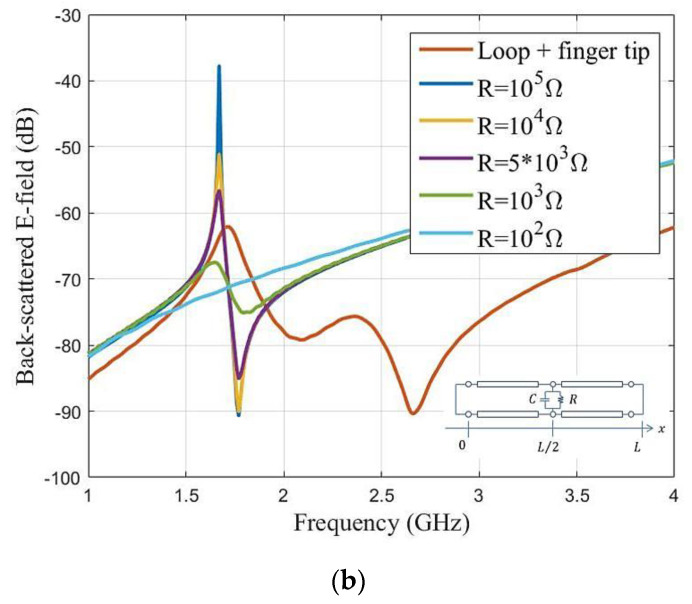
Comparison of backscattered E-field from the loop scatterer at different scenarios. (**a**) Capacitive effect in relation with Figure 8, (**b**) Parametric study on the introduction of a resistor R in parallel to consider the attenuation effect due to the presence of the finger.

In order to evaluate the performance of the introduced model and to justify the capacitive effect linked to the presence of the finger in contact with the loop, simulations were carried out on CST. Figure 8 shows the case where the fingertip is in contact with the loop (Figure 8a), as well as the case closer to the model (Equation (3)) where a capacitor (lumped element) was introduced in the middle of the loop (Figure 8b). Figure 9a presents the comparison of backscattered E-field from the scatterer at three different situations. The curve corresponding to loop alone (yellow curve in Figure 9a), i.e., without any influence of any external factors was used as the base curve for the comparison.

A second simulation was performed by placing the CST voxel model for the hand on top of the resonator (red curve in Figure 9a) to replicate the touch event scenario (Figure 8a). It was possible to extract the resonant frequency $f_{r}$ in the case of the finger loop and use Equation (4) to calculate the corresponding capacitance value *C*. The extracted value of *C* was 0.77 pF.

Then, for the third stage, a lumped element was created with the obtained capacitance value *C* = 0.77 pF) and placed between the coplanar lines (see Figure 8b). The observed backscattered response is also plotted in the Figure 9a (blue curve Figure 9a).

We could see that both configurations (touching the resonator with voxel model and the simulation with lumped element) created an exact 1.42 GHz shift in the resonant frequency to the lower side of the spectrum. Indeed, the resonance frequency dropped from 3.09 GHz (loop only) to 1.67 GHz when the finger was considered (both by the model and in full-wave simulation). We also observed a significant reduction in the amplitude of the back-scattered signal compared to loop alone (yellow curve of Figure 9a). The difference between the amplitude of backscattered signal, when the hand was in contact with the tag (red curve of Figure 9a), and the effect of the lumped element (blue curve in Figure 9a) was due to the fact that the losses were not included in the lumped element model. A parallel resistor R could be added to better match the effect of the finger; corresponding results are plotted in Figure 9b. It can be seen that, to get closer to the value obtained in full-wave simulation (red curve of Figure 9b), a parallel resistor with a value between 1 and 5 kΩ must be added.

## 5. Experimental Validation and Discussion

### 5.1. Prototype

**Figure 10:**
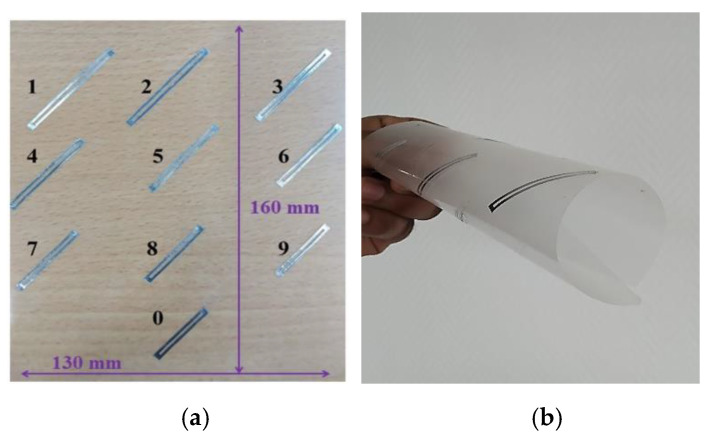
Proposed label: (**a**) Fabricated Chipless tag; (**b**) Image of fabricated tag in rolled form.

Three labels comprising ten loop scatterers distributed in the band 3 GHz to 5.6 GHz (geometrical lengths are given in Table 1) were realized by cutting aluminum tape (0.1 mm thickness). A slot with g = 1 mm, metallic width W1 = 1 mm, and W2 = 2 mm (Figure 4) were chosen for all resonators to facilitate the realization process. The resonators were then pasted on a thin PET substrate with a dielectric constant *ε_r_* = 2.6 and a loss tangent tanδ = 0.006 with 100 µm thickness and dimensions of 160 × 130 mm^2^. The resonators were arranged as represented in Figure 10 (the term digit 10 used in the article for the digit 0 in the Figure 10) to compose a decimal numeric keypad. The scatterers were oriented at 45° to achieve a compact design but were read in co-polarization. Each scatterer corresponded to a decimal digit which is indicated in Figure 10a.

### 5.2. Measurement Results: Coding

**Figure 11:**
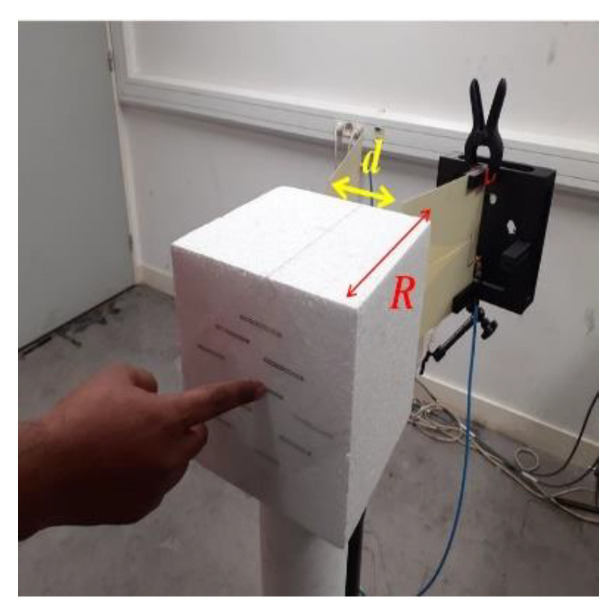
Measurement setup for the identification and touch sensing.

**Figure 12:**
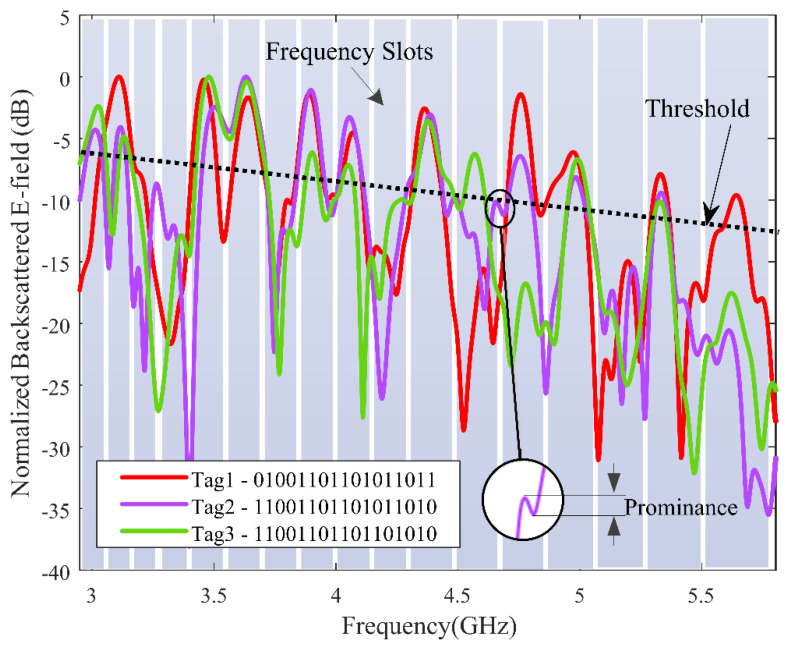
Measured frequency response of the fabricated tags with its ID.

The measurements were carried out in bi-static mode with co-polarization configuration in a normal office environment (see Figure 11). The label was positioned R = 27 cm away from low-cost printed vivaldi antennas whose phase centers were separated as d = 4 cm from each other (incidence of 9.6°). The distance of separation was defined by considering real-life scenarios, such as railway ticketing, museums, and parking, where the wireless keypad could be a potential candidate. The antennas are connected to an FCC compliant low-cost chipless RFID reader [21] interfaced to a laptop. An empty measurement was performed (no tag, no hand) and subtracted from the tag measurements to remove potential reflections from the setup. Time gating was also applied to the raw signal to filter out the contribution from potential reflections arising from real environment [31]. The backscattered fields from the three realized tags were measured for the frequency position coding for identification; the working frequency band (3 GHz to 5.6 GHz) was divided into 17 frequency slots. The slots were non-uniform to account for the decrease of the quality factor of the resonators with respect to the increase in frequency. The frequency slots are represented in Figure 12 with a width from 110 MHz to 250 MHz for lower to higher frequency.

The measurement results of the three tags as well as their respective ID are presented in Figure 12. In real environment, the data were subject to noise and the presence of ripples made decoding difficult. From this example we saw that a strategy must be employed to retrieve the correct code. The first action to be taken was to select peaks based on peak prominences in order to discard secondary peaks (ripples), as represented by the inline of Figure 12. In a second step, a threshold could be applied to select the peaks with a maximum height. The spectral energy distribution of the incoming signal was not uniform such that the threshold had to be chosen to decrease with frequency, as represented in Figure 12. Applying this two-step strategy, the identifiers were well-decoded for the three labels even in the office environment, with a single-layer tag and a low-cost reader. The coding capacity for 10 peaks distributed among 17 frequency slots [32] was given by calculating the total number of combinations, which can be obtained as $\left(\begin{array}{c} 17\\ 10 \end{array}\right)=19,448$; base 2 logarithm of the total combination value will give the bit, which is given in Equation (3).

$$C=\log_{2}\left[\left(\begin{array}{c} 17\\ 10 \end{array}\right)\right]=14.2\;\mathrm{bit}.\;$$

**Figure 13:**
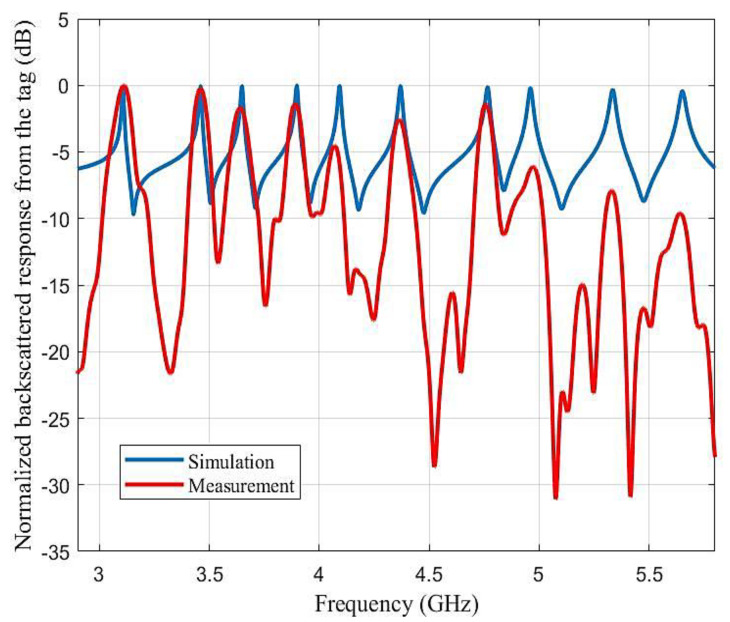
Simulation and measurement comparison of backscattered response from Tag 1 (Figure 12, red curve). Measurements were taken for the chipless reader, as discussed in [21].

A comparison study of the simulated backscattered response and measured backscattered response was completed to see the impact on the resonant frequency and Q factor. The results are shown in the Figure 13. We were able to achieve an excellent Q factor of 322 at the lowest resonant frequency and 170 at the highest resonant frequency in simulation. The results show a good compromise between the simulation and measurement. However, we also observed differences, as the reader distorts the acquisition due to its operating mode and the post-processing used. Indeed, the reader sends a short pulse which means that the spectrum of the signal was not constant in frequency (which is achieved for the simulation). Another comparison could have been obtained with the simulation if the power of the emitted pulse was considered to normalize the measured response; however, the idea here was to show the raw signal that we measured and that is used in the rest of the paper to retrieve both the identifier and the user’s interaction with the keyboard. A second important difference between the two signals is the temporal windowing used in the reader, which reduced the duration of the signals and therefore reduced the frequency resolution. This is why the peaks related to the resonance frequencies were much wider than those obtained in simulation. However, it can be seen from this comparison that the measured signal contains precisely the resonance frequencies and that they are all clearly recoverable by post-processing.

### 5.3. Measurement Result of Touch Gesture

**Figure 14:**
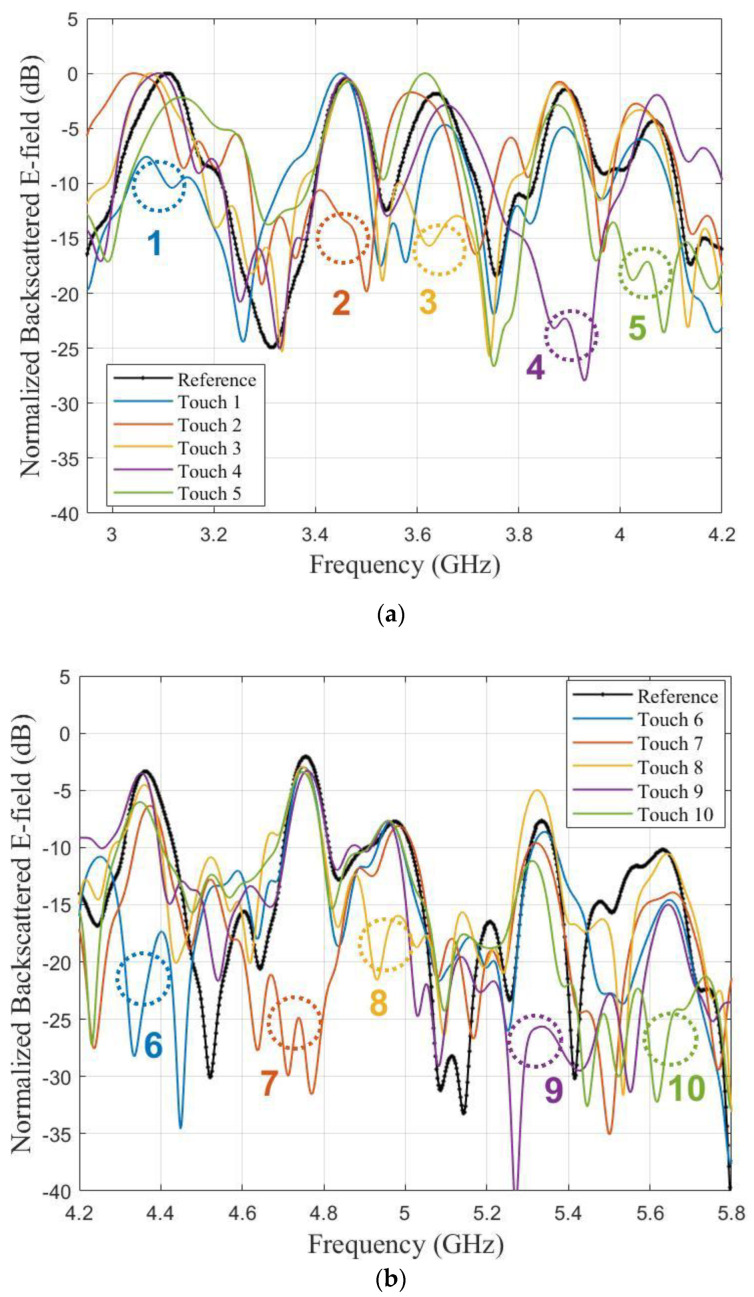
Measured backscattered E-field (in real environment) of the keypad for finger touching: (**a**) Digit 1 to 5; (**b**) Digit 6 to 10.

Tag 1 in Figure 12 was used to demonstrate the touch-sensing capability. The measurement setup was the same as the one represented in Figure 11. Each scatterers of the label were touched and corresponding backscattered responses were measured, which is then compared with the un-touched response. For readability, the touch gesture results are presented separately in Figure 14a (resonators n°1 to n°5 from 3.1 GHz to 4.2 GHz) and in Figure 14b (n°6 to n°10, 4.2 GHz to 5.8 GHz). In all cases, the presence of the finger cancelled the appropriate resonance with an average magnitude reduction of 15 dB. The presence of the finger had a non-negligible impact on the neighbouring peaks (increased by +2.34 dB compared to reference for curve touch 4 at 4.1 GHz) but it had a localized effect on the signature and did not undesirably suppress the response of the surrounding resonators. The peaks of the untouched resonators remained clearly visible and were less perturbed than the resonator touched by the finger.

### 5.4. Repeatability

**Figure 15:**
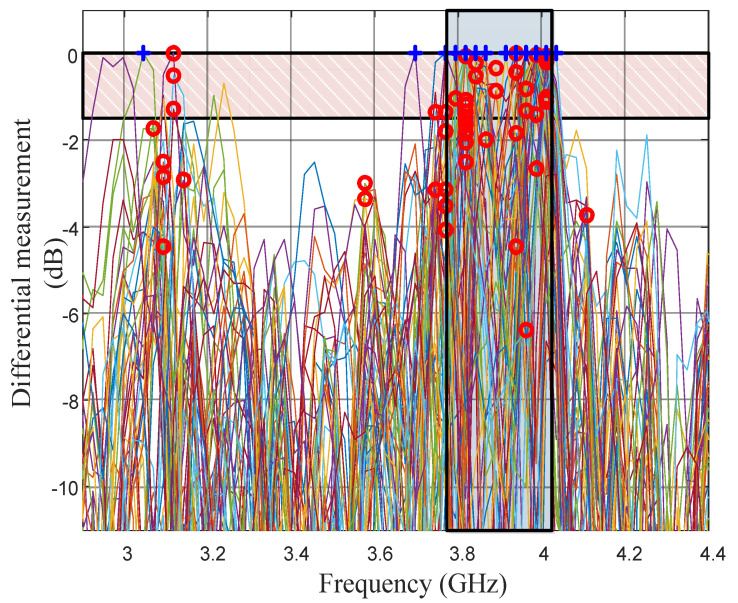
Maximum values of the relative measurement of the 4th scatterer for 50 sequences.

A series of 50 identical measurements were carried out to evaluate the repeatability of the approach. For each sequence, an initial measurement was taken without key pressing and then a second measurement was taken while pressing the digit n°4. The two measurements were then subtracted (relative measurement) which resulted in a maximum at the resonant frequency of resonator n°4. The maximum of the relative measurements (normalized for visibility) are represented in Figure 15. To determine if the detection is valid, the simplest approach was to select the maximum of the differential measurements (blue plus signs in Figure 15, which are discretely distributed due to the 25 MHz frequency resolution of the reader) and to assess that the point is in the frequency slot corresponding to resonator n°4 (3.9 GHz ± 125 MHz). This simple approach provided a read rate of 86% which may be insufficient for real applications. Inspecting the results of the differential measurement showed that, when false detection occurred, a peak was still present in the proper slot (resonator n°4), but a second peak of comparable height also appeared for another frequency, which may be due to the modification of the structural mode (non-resonant contribution of the finger).

**Figure 16:**
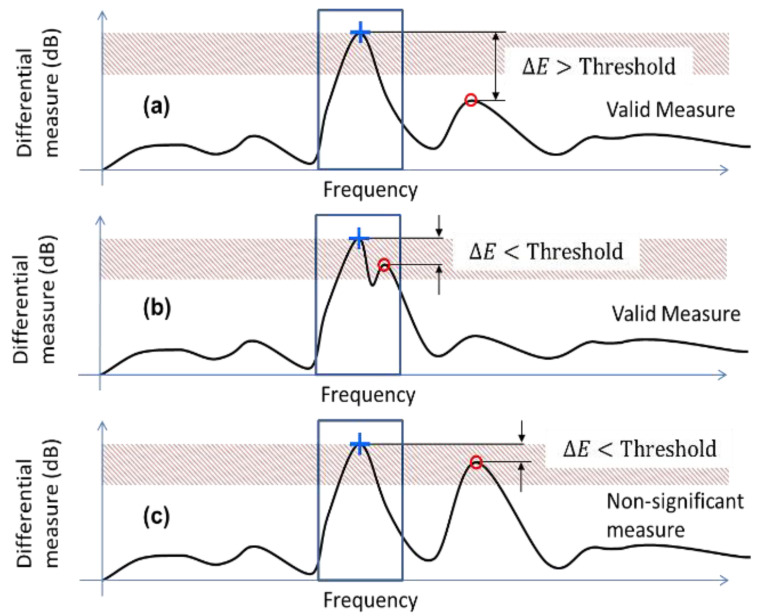
Conditions for ripples removal and valid detections: (**a**) Two peaks appear at two separate frequency slots with apex difference higher than threshold value. (**b**) Two peaks appear at same slots with apex difference less than threshold value. (**c**) Two peaks appear at two separate frequency slots with apex difference lower than threshold value.

A more robust technique was to select the maximum of the two major peaks (blue plus and red circle sign in Figure 15) and to compare them to see if one was dominant compared to the other. If one peak was dominant, i.e., superior to a threshold value compared to second peak (Figure 16a), the measure was considered valid and the slot corresponding to the resonant frequency was selected. If the two peaks were comparable and are in the same frequency slot (presence of ripples in the vicinity of the main peak), the measure was considered valid and the frequency slot was selected (Figure 16b).

If the two peaks were comparable and were in two different frequency slots (Figure 16c), we consider it impossible to decide if the key was pressed and the measure was discarded. In this manner, it is possible to differentiate non-significant measures (the key is not well detected and the measure has to be repeated) from false detection. A threshold of 1.5 dB for the previous set of data led to a detection rate of 82% with 18% discarded results and no false detection, which seemed to be more robust from a practical point of view.

## 6. Conclusions

The idea of decimal numeric keypad with unique ID using chipless RFID technology is put forward. The system used a two-stage approach to do its functionality. In the first stage, it used a radar technique to identify the label ID and, in the second stage, the perturbation was caused by the human hand to the fields associated with the RFID label to capture the touch event. Three tags, each containing 10 half-wavelength loop resonators, were used to study the feasibility of the identification step. Each resonator was designed in such a way to facilitate high Q factor to increase the coding capacity and high RCS to reduce the environmental effect. We were able to achieve a maximum Q factor of 322 at the lowest resonant frequency with good agreement between the simulation and measurement results compared to the previous works. An analytical model was developed to study the electrical behavior of the hand touch and a comparative study with the simulation results was used to verify the analytical model. The validity of the approach is tested both in simulation and measurements results. A repeatability study in the real environment was conducted in order to verify the reliability and robustness of the approach and a 82% detection rate was obtained with zero false detection. The detection rate was slightly lower for the real-world implementation but sufficient to prove the concept. We could achieve an excellent false detection rate of 0% which would help to improve the total system performance because successive measurements could be used to decrease the false detection rate. If the chipless reader read the tag response in microseconds, there would be less impact on the user experience and the limitation of the detection rate to a certain percentage would be covered. All measurements were taken in real time with a low-cost setup. With this system, we were able to produce unique ID keypad which can detect the human touch information with high accuracy without any complex computation techniques or algorithms. The labels used for the study was compatible with mass fabrication techniques. Further studies could be carried out to improve the system performance and imitate the real-world scenario by introducing various objects close to the wireless keypad. In terms of application, this system could be implemented as an alternative to current ATM and other dual security systems where the secure part corresponding to enter the pin code could be accomplished with a personal label that could be the card itself.

## Figures and Tables

**Table 1:** Length and resonant frequencies of 10 scatterers for three tags.

Scatterer	Tag 1		Tag 2		Tag 3	
	L (cm)	F (GHz)	L (cm)	F (GHz)	L (cm)	F (GHz)
1	4.2	3.1	4.4	3.0	4.4	3.0
2	3.8	3.5	4.2	3.1	4.2	3.1
3	3.6	3.6	3.8	3.8	3.8	3.5
4	3.4	3.9	3.6	3.6	3.6	3.6
5	3.2	4.1	3.4	3.9	3.4	3.9
6	3.0	4.4	3.2	4.1	3.2	4.1
7	2.7	4.8	3.0	4.4	3.0	4.4
8	2.6	5.0	2.7	4.8	2.8	4.6
9	2.4	5.3	2.6	5.0	2.6	5.0
10	2.3	5.6	2.4	5.3	2.4	5.3

L—Length of the loop resonator; F—Resonant frequency.
